# Retos de la inteligencia artificial y sus posibles soluciones desde la perspectiva de un editorialista humano

**DOI:** 10.7705/biomedica.7242

**Published:** 2023-09-30

**Authors:** Diego M. López

**Affiliations:** 1 Facultad de Ingeniería Electrónica y Telecomunicaciones, Facultad de Ciencias de la Salud, Universidad del Cauca, Colombia Universidad del Cauca Facultad de Ingeniería Electrónica y Telecomunicaciones Universidad del Cauca Colombia

*“La convergencia de la inteligencia artificial (IA) y la salud ha dado lugar a un nuevo paradigma en la atención médica y la investigación biomédica. Las tecnologías de IA, como el aprendizaje automático y el procesamiento del lenguaje natural, están transformando la forma en que los profesionales de la salud diagnostican enfermedades, personalizan tratamientos y mejoran la gestión de datos clínicos (...).*

*En este editorial, exploraremos las aplicaciones de la inteligencia artificial en el campo de la salud, resaltando sus logros, desafíos y el camino a seguir”* ^1^ *.*

Bastaría solamente con ingresar, al sitio web de ChatGPT, la instrucción “Escribe una (sic) editorial en español de 1.000 palabras sobre el tema: aplicación de la inteligencia artificial en salud, incluyendo referencias para un journal (sic) científico en biomedicina” y mi objetivo de preparar este editorial podría haberse cumplido.

Seguramente para la mayoría de los lectores, el uso de ChatGPT para elaborar este artículo podría haber pasado inadvertido y la calidad del contenido no sería discutida. El artículo estaría bien escrito, estando incluso sustentado por referencias de la revista *Nature*, aunque no fuesen las más recientes y relevantes, o incluso, inexistentes en otros casos. Sin embargo, además de las afirmaciones y generalizaciones hechas sin una evidencia científica clara, ese potencial manuscrito escrito por ChatGPT presentaría un sesgo importante al declarar principalmente las bondades de la inteligencia artificial, sin ahondar en los riesgos, limitaciones e inmadurez de la tecnología para llegar a impactar de manera efectiva los procesos de atención en salud humana. La inteligencia artificial tampoco hubiese sido capaz de proponer posibles soluciones a todos los retos planteados por ella misma.

En una investigación reciente, se llevó a cabo una revisión de alcance sobre el estado actual del conocimiento sobre la aplicación de la inteligencia artificial, en los países de bajos y medianos ingresos. Se describieron los principales proyectos, las contribuciones tecnológicas, los datos utilizados, el contexto de salud, el tipo de intervenciones de salud en los cuales se ha aplicado la inteligencia artificial, y los retos, brechas y posibles soluciones a los mismos, mediante el uso de esta tecnología ^1^. En esta revisión, se encontró que la mayoría de las investigaciones propusieron, de manera experimental, modelos de aprendizaje automático (*machine learning*) y, más recientemente, modelos basados en aprendizaje profundo (*deep learning*). Sin embargo, pocos estudios utilizaron los modelos en entornos clínicos reales.

En cuanto a la caracterización de las fuentes de datos, aquellos provenientes de los registros electrónicos de salud y las imágenes de radiología, son los tipos de datos más utilizados en los estudios experimentales de *machine learning*. La mayoría de los conjuntos de datos son pequeños, debido al alto costo de recopilar datos locales. Los grupos más grandes de datos corresponden a proyectos u organizaciones internacionales que recopilan información en los países de bajos y medianos ingresos, o proyectos en que se analizan datos abiertos con cohortes de pacientes de estos mismos países.

Con respecto al contexto de salud de las aplicaciones de inteligencia artificial en países de bajos y medianos ingresos, la mayoría de las intervenciones abordaron soluciones para salud materno-infantil, recién nacidos, niños y adolescentes. Otras iniciativas trataron de cáncer, salud mental y enfermedades cardiovasculares. La tuberculosis, el COVID-19, el HIV y las enfermedades transmitidas por vectores y parasitarias, completaron el grupo de intervenciones en enfermedades infecciosas.

En relación con el tipo de intervención, el objetivo principal de los estudios experimentales era el diagnóstico de enfermedades, seguido de la evaluación de la mortalidad y el desarrollo de sistemas de apoyo para la toma de decisiones. Otras investigaciones fueron de tipo observacional y abordaron temas como el análisis de los factores determinantes sociales de la salud, la distribución poblacional, la salud ambiental, el cambio climático y la prestación de servicios de salud.

Entre las brechas, los desafíos y las posibles soluciones para la implementación de la inteligencia artificial en los sistemas de atención médica de los países de bajos y medianos ingresos, se pueden destacar:

- *Calidad de los datos:* es un aspecto crucial para que las soluciones de inteligencia artificial respondan a las necesidades y al contexto para el cual han sido creadas. La calidad abarca criterios como la precisión, la coherencia, la integridad, la credibilidad y la actualidad del dato.

Al respecto, los algoritmos de inteligencia artificial deben probarse y evaluarse utilizando datos locales que cumplan con estos parámetros. Es por esto que revisten mayor importancia las regulaciones nacionales en la implementación de sistemas de información en salud, integrados a nivel nacional -como las historias clínicas electrónicas- pues son la fuente primaria para probar modelos de inteligencia artificial.
Esto también es válido para otras tecnologías en salud, como dispositivos médicos, sensores, aplicaciones móviles y datos de vigilancia en salud pública, los cuales mejoran los criterios de disponibilidad y diversidad de las fuentes de datos.
Un reto importante es garantizar la calidad de los datos. Para esto, se requiere invertir gran parte de los recursos de los proyectos de inteligencia artificial en salud, en la preparación y curaduría de los datos y, además, involucrar a expertos en datos y equipos multidisciplinarios con conocimientos y experiencia en el dominio de la salud.
El proceso de gobernanza incluye políticas de calidad de los datos para proporcionar conjuntos de datos certificados por organizaciones locales e internacionales, independientes y confiables. Un mecanismo de gobernanza puede ser la adopción de los principios FAIR (fáciles de encontrar, accesibles, interoperables y reusables).
La mejoría de la calidad implica el uso de herramientas métricas claras y estandarizadas para la gestión de los datos, el diseño conjunto de soluciones de inteligencia artificial (con usuarios, personal de la salud, pacientes y administradores clínicos), y la creación, el uso y la implementación de bases de datos de código abierto, como lo hace el proyecto MIMIC-IV ^2^.

- *Localización de los modelos*: otro de los desafíos importantes para el éxito de las soluciones de inteligencia artificial en salud es que los modelos sean creados con datos representativos del contexto en el cual serán utilizados.

La mayoría de los modelos de inteligencia artificial utilizados en los países de bajos y medianos ingresos, suelen construirse con datos de países de ingresos altos con diferentes contextos, preferencias culturales, protocolos médicos y características demográficas, lo que produce el llamado sesgo del contexto.
Para enfrentar estos desafíos, es necesario el compromiso de las partes y establecer alianzas estratégicas entre prestadores de servicios de salud, la academia y la industria en general. También, es necesario establecer los marcos reguladores, y las políticas para el uso controlado, seguro y abierto de los datos. Las intervenciones de inteligencia artificial deben priorizarse de acuerdo con la carga de la enfermedad en cada contexto de uso.

- *Marcos reguladores y legales:* las políticas y regulaciones son esenciales para implementar con éxito las soluciones de inteligencia artificial. Estas incluyen políticas de privacidad, seguridad, consentimiento informado, ética, confidencialidad y responsabilidad.

La gobernanza y el liderazgo locales son necesarios para promover y ejecutar estrategias de inteligencia artificial en el marco de las estrategias de salud digital, a nivel nacional, regional y local.
Para abordar estos desafíos, se recomienda hacer obligatorio el registro de las intervenciones basadas en inteligencia artificial, aprobar los protocolos de investigación y consentimiento informado ante los comités de ética, y ajustar las políticas locales a la regulación internacional, pero con el desarrollo de aspectos reguladores según el país.
Las políticas y los marcos legales de inteligencia artificial deben proteger a las personas contra comportamientos poco éticos. Asimismo, los gobiernos, los usuarios finales, los proveedores de atención médica y los desarrolladores de inteligencia artificial deberían compartir la responsabilidad en caso de su uso inapropiado.

- *Sesgo sistemático en los modelos:* otra de las limitaciones importantes en los modelos *de machine learning* es la imprecisión sistemática de las predicciones entregadas.

Un ejemplo común es el sesgo por raza que presentan algunos modelos. Por ejemplo, un algoritmo de *machine learning* concluyó erróneamente que los pacientes de raza negra, en una población en particular, eran más saludables que los de raza blanca ^3^. El sesgo se debe a que el algoritmo la asignó un valor importante a los costos de atención en salud, infiriendo que, como el grupo poblacional de raza negra recibió un menor gasto en servicios de salud, su estado de salud era mejor.
En una región como Latinoamérica, con numerosas comunidades indígenas y afrodescendientes que enfrentan fallas sistémicas en el acceso a los servicios de salud -debido a barreras geográficas, culturales y económicas- se vuelve imperativo abordar este desafío.
Un mecanismo para afrontar este sesgo sistemático es diversificar las fuentes de datos en términos de sexo, raza, factores étnicos y socioeconómicos, etc. Es claro que la diversificación de los datos y la recolección de conjuntos de datos locales de calidad, son tareas costosas y demoradas. En este sentido, la validación local de modelos probados anteriormente en otros contextos, puede proporcionar una solución a corto plazo mucho más práctica para abordar el sesgo de los modelos.
Sin embargo, esto requiere priorizar la inversión en infraestructura y capacidad para la validación local y la recalibración de los modelos ^4^. Además, en la gestión y prevención del sesgo, los sistemas de inteligencia artificial deben garantizar la transparencia de las fuentes de datos y algoritmos usados en la comprobación, la evaluación y la validación de los modelos.
Por lo tanto, se necesitan estrategias de ciencia abierta y liberación de datos, así como el fomento del avance de técnicas, para mejorar la interpretación y la explicación de los modelos de *machine learning*.

- *Creación de capacidades en inteligencia artificial*: los desafíos de la educación y la resistencia al cambio dificultan la comprensión, el uso, la formulación de políticas, la investigación y la innovación en tecnologías de inteligencia artificial.

Las recomendaciones frente a este reto incluyen el desarrollo de capacidades por medio de la academia, organizaciones y sociedades científico-gremiales, la capacitación y la retención de expertos locales, y la diversidad en la formación, en particular, enfocada en poblaciones vulnerables y comunidades en riesgo de verse afectadas por la inteligencia artificial (como el “sesgo sistémico” antes mencionado).
Los incentivos económicos y los modelos comerciales innovadores en torno a la recopilación y agregación de datos podrían dar un impulso a la creación de soluciones de inteligencia artificial.
La creación de capacidades incluye la adopción de estándares metodológicos, el diseño de soluciones, centradas en el humano, y la adopción de mecanismos de certificación.
Se requieren guías metodológicas para la generación de evidencia científica, como estudios clínicos aleatorizados para evaluar el impacto a largo plazo de las intervenciones basadas en inteligencia artificial.
En este sentido, existen iniciativas como las recomendaciones y guías de CONSORT-AI y SPIRIT-AI ^5^, DECIDE-AI ^6^, y las directrices de la Organización de Naciones Unidas (ONU), la Unión Internacional de Comunicaciones (UIT) y la Organización Mundial de la Salud (OMS), para respaldar estos desafíos.

- *Conectividad e infraestructura*: Los desafíos de conectividad e infraestructura de datos también son significativos. El mayor uso de las redes móviles ha mejorado la conectividad en los países de bajos y medianos ingresos, pero muchas áreas rurales carecen de acceso continuo a internet. La inversión en conectividad universal a internet es una prioridad, al igual que la disponibilidad de los registros electrónicos de salud y el acceso seguro a los datos de la historia clínica electrónica, que son problemas aún sin resolver en muchos países y regiones.

Los gobiernos, los prestadores de salud, las asociaciones profesionales y otros actores, deben promover la construcción de infraestructuras nacionales de salud digital, incluidas las plataformas de interoperabilidad y las plataformas para *machine learning* colaborativo, y para familiarizarse con vocabularios, terminologías y ontologías.

- *Recursos financieros y líneas directrices de incentivos:* la asignación de recursos financieros es otro desafío en los países de bajos y medianos ingresos para implementar tecnologías digitales. Muchos países dicen priorizar la salud digital, las tecnologías de inteligencia artificial y el aprendizaje automático, pero la asignación de recursos -escasos- no se lleva a cabo en la práctica.

Las soluciones para superar esto incluyen el establecimiento de agendas nacionales de investigación e innovación para las intervenciones de inteligencia artificial. En instituciones prestadoras de servicios de salud es crucial establecer una estrategia de negocios y valor en salud. Esto implica designar un líder especializado en inteligencia artificial que, basándose en una premisa de costo- efectividad, contribuya a alinear los incentivos tanto internos como externos en relación con la adopción de esta tecnología.

## Conclusiones

Existe una creciente masa crítica de investigación sobre el uso de la inteligencia artificial para mejorar las intervenciones de atención médica en los países de bajos y medianos ingresos.

Hay muchas otras áreas de aplicación que vienen desarrollándose, como el tratamiento personalizado de enfermedades, la analítica predictiva (en progreso de enfermedad y acciones proactivas), el descubrimiento de fármacos, el monitoreo remoto, el cumplimiento de los pacientes, la optimización de procesos y la eficiencia administrativa, etc. Pero, más allá del vertiginoso avance de la inteligencia artificial, hay un conjunto amplio de brechas que deben resolverse primero para una implementación responsable en la práctica clínica, y deben escalarse los proyectos incipientes, aún en etapas de experimentación y desarrollo piloto.

Se requiere mejorar la calidad de las fuentes de datos actuales y la capacidad para diseñar e implementar soluciones de inteligencia artificial construidas con datos de los contextos locales; abordar los sesgos sistemáticos y garantizar la generalización de los modelos. Además, es preciso crear e incentivar la adopción de políticas y reglas para garantizar la privacidad, la seguridad, la ética, la responsabilidad y la confidencialidad de los datos y, en últimas, aumentar la confianza sobre el uso de los modelos.

También, se necesitan entornos y políticas sólidas de salud digital. Esto implica contar con infraestructura tecnológica, conectividad, interoperabilidad de los sistemas de información, normativas para la producción de datos y validación de las soluciones de inteligencia artificial mediante estudios clínicos aleatorizados.

Más adelante, se requerirá la certificación de las tecnologías basadas en inteligencia artificial, así como garantizar la inversión y la sostenibilidad de los proyectos basados en este tipo de tecnologías, para lograr sistemas más eficientes, sin perder al paciente como centro de la atención en salud.

## Footnotes

1 Texto generado con ChatGPT (https://chat.openIA.com/) el 3 de agosto de 2023. Disponible en: https://shorturl.at/ADHPV
